# Application of triple quadrupole mass spectrometry for the characterization of antibody–drug conjugates

**DOI:** 10.1007/s00216-019-01699-0

**Published:** 2019-03-08

**Authors:** Malin Källsten, Matthijs Pijnappel, Rafael Hartmann, Fredrik Lehmann, Lucia Kovac, Sara Bergström Lind, Jonas Bergquist

**Affiliations:** 1 Department of Chemistry-BMC, Analytical Chemistry, Uppsala University, Box 599, 751 24 Uppsala, Sweden; 2 Recipharm OT Chemistry AB, Virdings allé 32b, 754 50 Uppsala, Sweden; 3 Department of Medicinal Chemistry, Organic Pharmaceutical Chemistry, Uppsala Biomedical Center, Uppsala University, Box 574, 751 23 Uppsala, Sweden; 4 Oncopeptides AB, Luntmakargatan 46, SE-111 37 Stockholm, Sweden

**Keywords:** Reversed-phase liquid chromatography, Antibody–drug conjugates, Drug-to-antibody ratio, Triple quadrupole mass spectrometry

## Abstract

Antibody–drug conjugates (ADCs) are an inherently heterogeneous class of biotherapeutics, the development of which requires extensive characterization throughout. During the earliest phases of preclinical development, when synthetic routes towards the desired conjugate are being assessed, the main interest lies in the determination of the average drug-to-antibody ratio (DAR) of a given batch as well as information about different conjugation species. There has been a trend in mass spectrometry (MS)–based characterization of ADCs towards the use of high-resolving mass spectrometry for many of these analyses. Considering the high cost for such an instrument, the evaluation of cheaper and more accessible alternatives is highly motivated. We have therefore tested the applicability of a quadrupole mass analyzer for the aforementioned characterizations. Eight ADCs consisting of trastuzumab and varying stoichiometries of Mc-Val-Cit-PABC-monomethyl auristatin E conjugated to native cysteines were synthesized and served as test analytes. The average DAR value and molecular weights (Mw) of all detected chains from the quadrupole mass analyzer showed surprisingly high agreement with results obtained from a time-of-flight (TOF) mass analyzer and hydrophobic interaction chromatography (HIC)–derived values for all investigated ADC batches. Acquired Mw were within 80 ppm of TOF-derived values, and DAR was on average within 0.32 DAR units of HIC-derived values. Quadrupole mass spectrometers therefore represent a viable alternative for the characterization of ADC in early-stage development.

**Electronic supplementary material:**

The online version of this article (10.1007/s00216-019-01699-0) contains supplementary material, which is available to authorized users.

**Figure:**
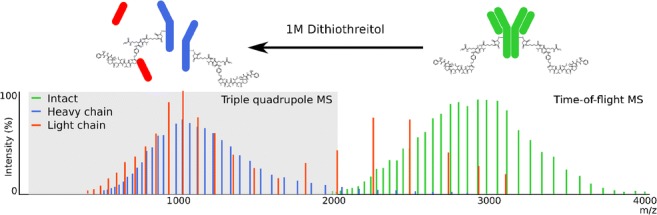
Graphical abstract

## Introduction

Antibody–drug conjugates (ADCs) have, owing to their epitope targeting ability, become highly popular contesters for targeted drug delivery, especially in oncology [1–3]. Due to their inherent heterogeneity, there is a plethora of options available for their characterization [4, 5]. In recent years, the trend in ADC analysis has been moving towards native mass spectrometry (MS) characterization using high-resolving MS (HRMS) instruments for drug-to-antibody ratio (DAR) determination [5–9]. The high resolution however comes at exorbitant cost and is thus not an option for all analysts in the ADC field.

Quadrupole mass analyzers, such as triple quadrupoles, are common for quantification of small molecules [10] and are commonly used for the quantification of unconjugated payloads in ADC samples [11]. Many laboratories working with ADCs therefore have ready access to at least one quadrupole mass spectrometer. Thus, we decided to evaluate the triple quadrupole’s potential for the characterization of the ADCs themselves, mainly focusing on DAR determination. Although scarce, there is literature precedent for the use of quadrupoles for intact protein analysis [12–18], and its application to the analysis of monoclonal antibodies (mAbs) has been proposed [12, 15, 16] with the majority deploying triple quadrupole mass analyzers. However, quadrupoles are not commonly considered a viable option for ADC characterization [5, 19], and to the best of our knowledge, this is the first time a triple quadrupole mass analyzer is evaluated for the analysis of complex ADC mixtures. Compared with therapeutic proteins such as mAbs, ADC samples commonly contain a mixture of different conjugation species, which differ in their DAR. Partially overlapping peaks in the *m*/*z* spectrum could potentially influence the relative peak areas of deconvoluted LCs and HCs which directly influence to the accuracy of acquired average DAR values. Consequently, it is necessary to confirm that the resolution of a quadrupole mass analyzer is sufficient to distinguish the individual molecular species.

At acidic pH, intact antibodies or ADCs produce ions of 2000–5000 *m*/*z* in electrospray ionization (ESI)-MS [20]. Previous publications have primarily focused on intact antibodies using quadrupoles or ion traps with a mass range of up to 3800 *m*/*z* [12, 21]. ADCs can very easily be cleaved into their light chains (LCs) and heavy chains (HCs) by means of disulfide reduction, which shifts the charge envelope of interest in ESI to a range of 800–2500 *m*/*z*. Reduced ADCs therefore produce ions which are readily detectable by most quadrupole mass analyzers; however, the number of analytes increases with this approach.

In this publication, a triple quadrupole mass spectrometer with a mass window up to 2040 *m*/*z* was evaluated for average DAR and molecular weight (Mw) determination of cysteine-linked trastuzumab–vcMMAE ADCs with eight distinct average DAR values. For comparison, analyses were also performed using a time-of-flight (TOF) mass analyzer and hydrophobic interaction chromatography with ultraviolet and visual light detector (HIC-UV/Vis).

## Materials and methods

All chemicals used were of analytical grade and were purchased from Sigma-Aldrich with the following exceptions: trastuzumab (Carbosynth), Mc-VC-PABC-MMAE (abcr), dithiothreitol (Panreac Applichem), acetonitrile (Carlo Erba), ammonium sulfate (Merck), potassium phosphate dibasic (Merck), and potassium phosphate monobasic (VWR).

### Sample preparation

ADC samples were generated according to an established procedure [22] by dithiothreitol (DTT)- or tris(2-carboxyethyl)phosphine (TCEP)-mediated reduction of interchain disulfides of commercially obtained trastuzumab, followed by the addition of an excess amount of Mc-VC-PABC-MMAE (vcMMAE) and subsequent desalting by means of ultracentrifugation. All protein solutions were diluted to a concentration of 1 mg/mL. Prior to reversed-phase (RP)-MS analyses, all samples were deglycosylated by the addition of 3 μL PNGaseF (500 units/mL) to 50 μg of protein, followed by 24-h incubation at 37 °C and subsequent reduction using 3 μL of 1 M DTT at room temperature for 30 min.

### Quadrupole RP-MS

Triple quadrupole mass analyses were performed using a Waters Xevo TQ-S Micro MS equipped with a Z-spray ESI source connected to a Waters Acquity UPLC system. A Waters Acquity UPLC Protein BEH C4 column 2.1 mm × 50 mm with 300-Å pores and 1.7-μm particles with a Waters C4 BEH Vanguard precolumn was used for all analyses. Mobile phases consisted of 0.5% formic acid in water or acetonitrile and the gradient went from 25 to 85% organic solution over 8 min at a flow rate of 0.4 mL/min. The gradient included column equilibration at 25% B for 1 min both at the beginning and at the end of the gradient. Column temperature was set to 50 °C. Five micrograms of the reduced forms of trastuzumab and ADCs was injected in triplicates, and spectra were collected over the mass range 500–2040 *m*/*z* calibrated at 0.75 *m*/*z* FWHM unit resolution. The triple quadrupole was run in normal scan mode (MS1) where the ions are separated in the first quadrupole and the last two quadrupoles are used mainly as ion guides, allowing the transmission of ions over the entire mass range selected to pass through all three quadrupoles. Capillary voltage was set to 3.0 kV and cone voltage to 40 V, source temperature was 150 °C, desolvation gas and temperature were set to 1200 L/h and 650 °C, respectively, and collision energy was set to 3 V to help ensure the complete desolvation of the analytes before they reach the detector.

### Time-of-flight RP-MS

For comparison, all samples were also analyzed using Agilent’s QTOF 6550 iFunnel LC/MS equipped with an ESI source connected to Agilent’s 1290 Infinity II LC system. The same column, mobile phases, and reversed-phase liquid chromatography (RPLC) gradient were applied as in quadrupole RP-MS analyses. The column temperature was set to 60 °C. Five micrograms of the reduced forms of trastuzumab and ADCs was injected in triplicates, and spectra were collected over the mass range 800–3200 *m*/*z*. The TOF was run in MS mode and calibrated for high mass range of up to 10,000 *m*/*z* and a resolution of 28,000 FWHM at 2100 *m*/*z* at calibration. Gas temperature was set to 250 °C, drying gas was 16 L/min, and the nebulizer was set to 40 psig. Sheath gas temperature was set to 350 °C and the flow rate was set to 11 L/min. Capillary voltage was 4 kV, nozzle voltage was 1 kV, fragmentor was 380 V, collision energy was kept at 10 V, and the RF of octopole 1 was 750 V.

### HIC-UV/Vis analyses

Fifty micrograms of trastuzumab or ADC was mixed with 5 μL 50:50 (mobile phase A:mobile phase B) before analysis. Sixteen microliters of sample was injected in triplicate for each batch and run on a Waters Protein-PAK Hi Res HIC 4.6 × 100 mm column with a 10-min gradient from 0 to 100% mobile phase B (62.5 mM phosphate buffer pH 6.8, 5% isopropanol) followed by 10-min re-equilibration with 100% mobile phase A (1.25 M ammonium sulfate in mobile phase B). Flow rate was set to 0.7 mL/min and chromatograms were collected at 220 nm, 280 nm, and 250 nm.

### Data evaluation

Mass spectra were extracted over all chromatographic peaks and combined into one spectrum (containing both LC and HC signals) followed by deconvolution using the MaxEnt1 algorithm in MassLynx (Waters) for triple quadrupole data and using the protein deconvolution function in MassHunter (Agilent) for the TOF data. DAR was calculated using weighted peak areas as described elsewhere [1].

## Results and discussion

### Molecular weight assignment

**Fig. 1:**
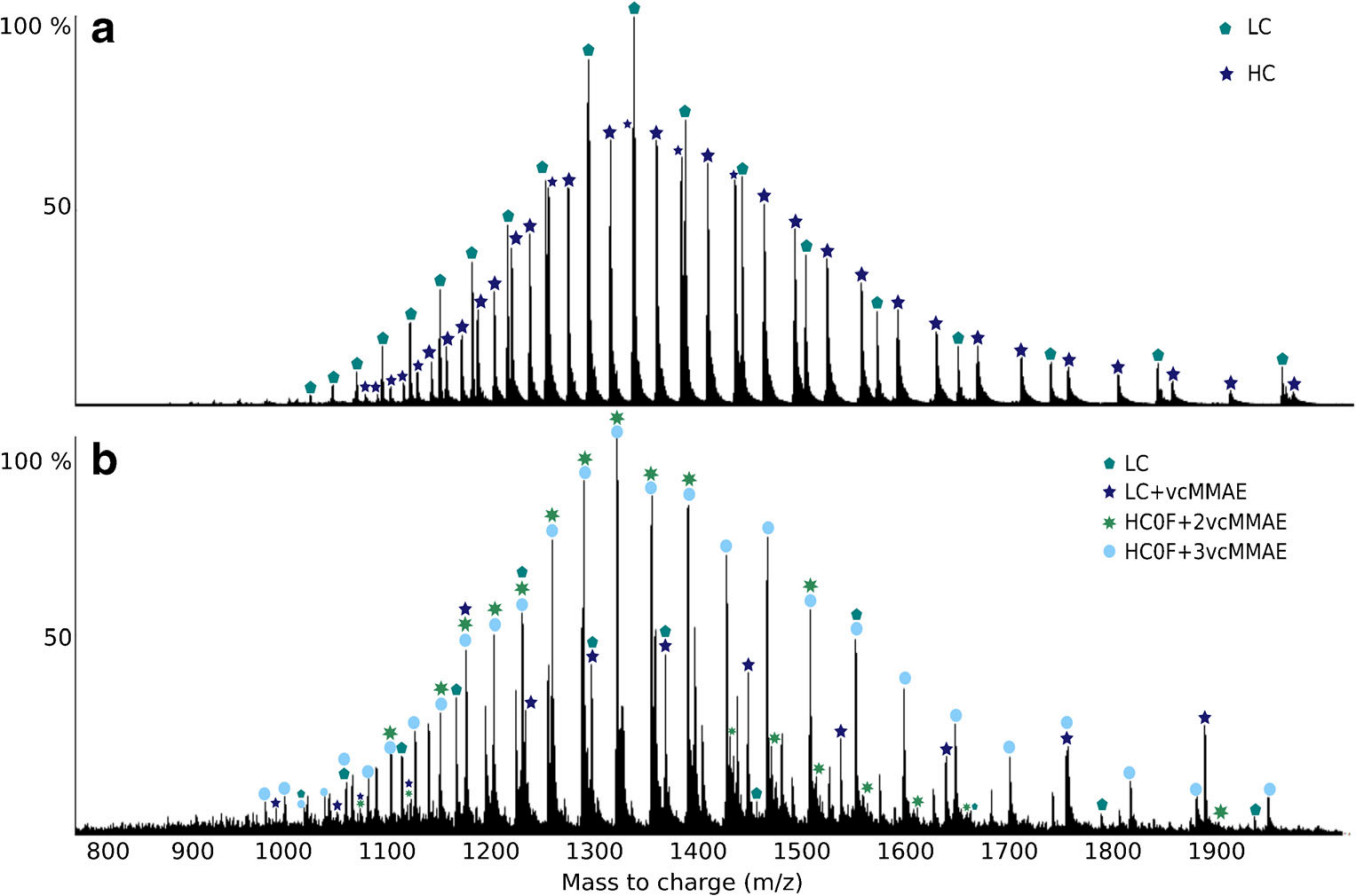
Mass spectra of LCs and HCs for unconjugated trastuzumab (**a**) and an ADC with DAR 7 (**b**) as detected on the triple quadrupole mass analyzer. Each peak has been assigned to its corresponding chain. Some peaks in the middle of the charge envelopes have very similar *m*/*z* and are difficult to assign to their originating chain; however, the majority of peaks can be unambiguously assigned even for the ADC sample

**Fig. 2:**
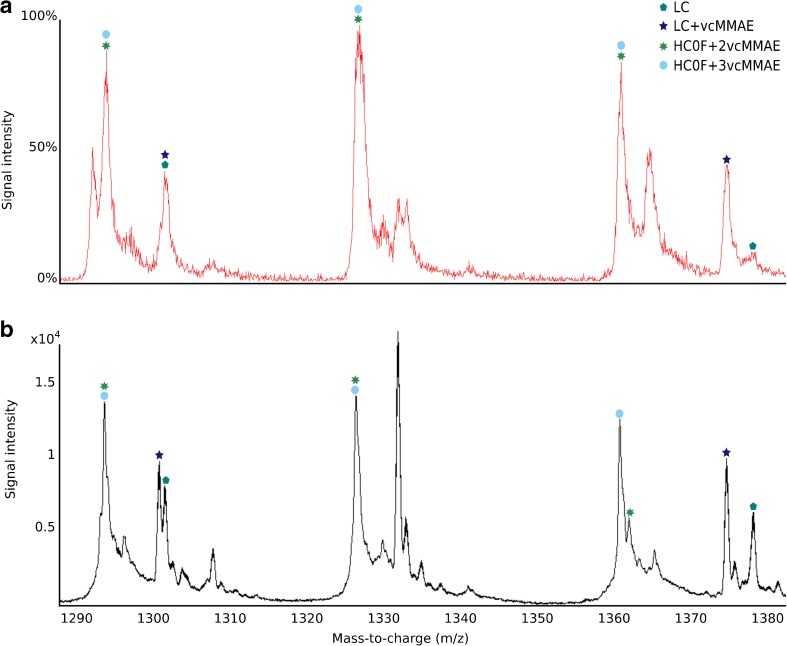
Zoomed in *m*/*z* spectra centered around the most abundant peaks of the overall spectra from the triple quadrupole MS (**a**) and TOF MS (**b**) of an ADC with an average DAR of 7. Peaks originating from both unconjugated and conjugated LC and HC can be observed clearly in both spectra although the TOF spectrum displays sharper and better resolved peaks

Previous studies have already shown that quadrupole mass analyzers can be used to obtain high-accuracy deconvoluted masses of intact Fab and Fc fragments of mAbs [15], and it has even been possible to resolve different glycan isoforms on intact antibodies [12]. The majority of precedent research on intact protein detection on quadrupole mass analyzers, including the current study, has utilized triple quadrupole mass analyzers either in multiple reaction monitoring (MRM) mode [14, 17, 18] or in normal scan mode [15, 16]. In the latter case, the first quadrupole in the triple quadrupole instrument was used for the ion separation and the last two quadrupoles as ion guides. Consequentially, single quadrupole instruments could also be a viable option. However, whether the use of a single quadrupole MS would lead to significant differences in regard to mass accuracy has not yet been investigated. In the aforementioned cases, however, the analyte consisted of a single protein. In ADCs, the covalently attached linker and drug moiety commonly shifts the mass by about 1–2 kDa, and differences in DAR species in a single batch create a heterogeneous mixture. Certain conjugated or unconjugated LCs and HCs have very similar *m*/*z* values in the center of their charge envelopes (see Fig. 1) which could potentially influence their deconvoluted mass. However, several peaks detected in the TOF data could also be unambiguously assigned in the quadrupole spectrum (see Figs. 1 and 2) for an ADC with an average DAR of 7. In this study, we will proceed to verify whether the unit resolution of a quadrupole mass analyzer is sufficient to accurately deconvolute each distinct conjugate chain over several ADC batches.

**Fig. 3:**
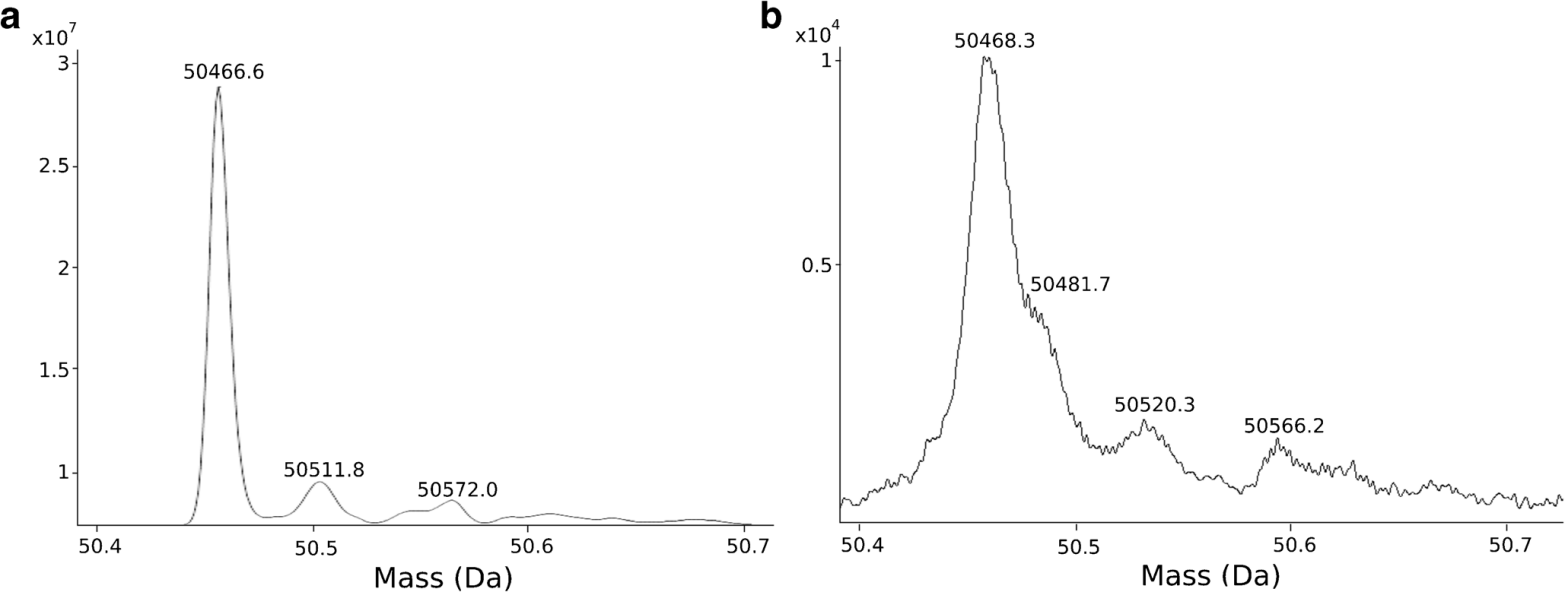
Deconvoluted mass for the HC with one conjugated linker–drug moiety (HC+1) acquired by the triple quadrupole (**a**) and TOF mass analyzer (**b**) for an ADC with an average DAR of 1.5, showing one major peak at 50,467 Da and two minor peaks at 50,512 and 50,572 Da in the triple quadrupole. The main peak can be confirmed with the TOF spectrum

Despite reducing the ADC into its LC and HC components, a minor portion of the charge envelope will still be outside the range of the triple quadrupole (Electronic Supplementary Material (ESM) Fig. S1). Before determining the DAR, it is hence necessary to assess whether this loss significantly impacts the deconvoluted mass, irrespective of the degree of conjugation. Comparing the derived masses of LCs and HCs, with and without conjugation, obtained from an ADC with an average DAR of 1.5 on the triple quadrupole mass analyzer with those acquired with a TOF mass analyzer, all values were in surprisingly good agreement (Table 1). Tables displaying the corresponding values for the remaining ADC batches can be found in ESM Tables S1–S7. Relative standard deviation (RSD) from triplicate injection is displayed for each chain showing that all masses are within 24-ppm accuracy between injections for the triple quadrupole mass analyzer. As predicted, RSD between injections on the TOF mass analyzer was lower at a maximum of 14 ppm. Furthermore, acquired Mw on the TOF mass analyzer (within 47 ppm) are closer to elsewhere reported values for unconjugated and deglycosylated LCs and HCs of trastuzumab [23] than on the triple quadrupole mass analyzer (within 77 ppm). However, the mass accuracy obtained on the triple quadrupole mass analyzer indicates that it can be used with ease to verify the success rate of conjugation for new ADC batches. The difference between the two mass analyzers is within 2.1 Da (within 80 ppm) for all detected ADC chains, which is lower than most mass shifts associated with post-translational modifications (PTMs). It may therefore be possible to monitor changes in the PTM profile over time/between batches by the means of a quadrupole mass analyzer; however, accurate identification of specific PTMs is difficult due to limited mass accuracy combined with the broad peaks from overlapping isotopes. In Fig. 3, a second peak at a mass shift close to that of an acetylation (+ 42.0 Da) can be observed. However, deamidation (− 1 Da) and other small-mass-shift-PTMs are outside the reach of the triple quadrupole mass analyzer.

**Table 1:** Evaluation of Mw of LC and HC species for an ADC with an average DAR of 1.5

ADC chain	Quadrupole mass analyzer		TOF mass analyzer		Difference between techniques (Da)
	Mw (Da)	(RSD^b^)	Mw (Da)	(RSD^b^)	
LC	23,438.3	(1 ppm)	23,440.1	(3 ppm)	1.9
LC+1^a^	24,755.1	(18 ppm)	24,756.0	(3 ppm)	0.9
HC	49,149.0	(1 ppm)	49,150.7	(1 ppm)	1.7
HC+1^a^	50,466.1	(2 ppm)	50,467.3	(3 ppm)	1.8
HC+2^a^	51,782.9	(24 ppm)	51,784.9	(−)	2.1
HC+3^a^	53,101.1	(0.3 ppm)	53,101.3	(14 ppm)	0.1

^a^The number annotates the number of conjugated one payload-linker moiety to each chain

^b^RSD of triplicate injections

### Sensitivity

**Fig. 4:**
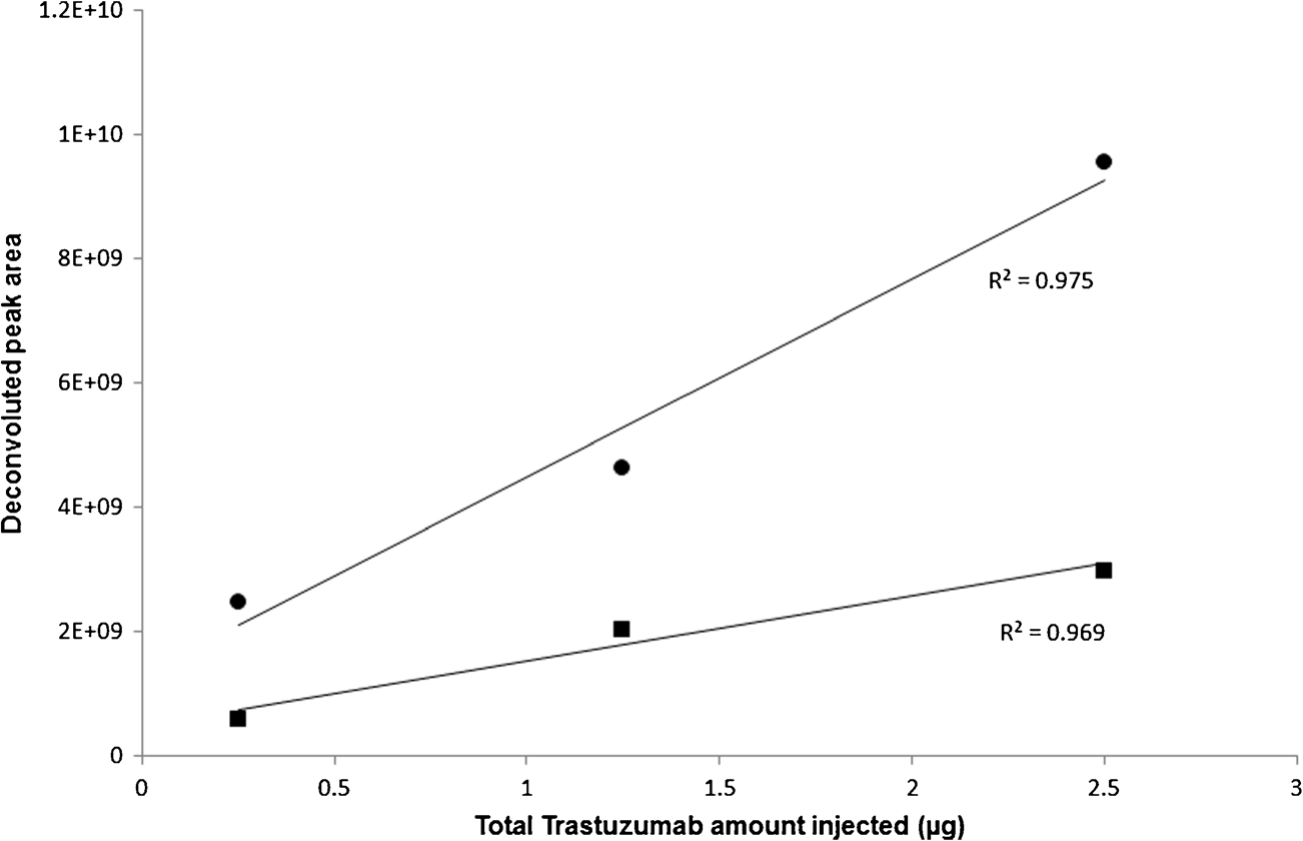
Linearity and sensitivity of the triple quadrupole mass analyzer. Linear increase in deconvoluted peak area over a concentration range from 0.25 to 2.5 μg of total amount injected unconjugated trastuzumab of chromatographically co-eluting LC (square markers) and HC (circle markers) with a *R*^2^ value of 0.97 for both chains

The ion source settings were optimized for optimal desolvation, resulting in high intensity spectra for as little as 0.25 μg of injected protein on the triple quadrupole mass analyzer. Damen et al. [12] have previously evaluated the linearity of the TIC response of a triple quadrupole mass analyzer for the quantification of intact antibody where a linear response up to 200 μg/mL could be observed. In order to assess whether a linear correlation could be extended to free LCs and HCs, trastuzumab was here reduced to its LCs and HCs and injected at increasing concentration. Monitoring the deconvoluted peak area of unconjugated LC and HC from 0.25 to 2.5 μg of total amount injected antibody (Fig. 4), a fairly linear response could be observed. Thus, quadrupole mass analyzers show potential for the quantification of individual antibody chains even without peak separation in the TIC.

In RPLC-UV detector–based quantification of LCs and HCs of mAbs/ADCs, base peak resolution in the total ion chromatogram (TIC) is commonly achieved by the use of trifluoroacetic acid (TFA) [24, 25]. However, to avoid the use of TFA, due to its known ion suppression effects [18, 26], and to limit the analysis time, the gradient applied in this study was optimized in regard to total run time and some degrees of chromatographic overlap between peaks of different chains were considered acceptable.

### Drug-to-antibody ratio

As has been reported previously [27], the average DAR value for a given ADC production batch may vary up to ± 0.3 DAR units between analytical techniques and a variability of 0.01–0.4 DAR units between different injections of the same ADC for a specific technique. For the triple quadrupole mass analyzer, DAR varied between injections up to 0.5 DAR units for one batch of ADCs, but was below 0.2 DAR units for all other batches, and on average within 0.32 DAR units from HIC-UV/Vis-derived values and was therefore in good agreement with previous findings. It is worth noting that the DAR value for the HIC data is calculated based on the intact form whereas the MS-derived values are calculated separately for LC and HC before summed to the total value (as described elsewhere [1]). In the event that chains of a certain degree of conjugation are partially lost on the column or suppressed in the ion source, this can impact the average DAR value and is most likely the main reason behind the discrepancy between the HIC- and MS-derived values. Standard deviation of average DAR between consecutive injections of the same ADC on the triple quadrupole mass analyzer is high compared with that on the TOF mass analyzer (below 0.15 DAR units) and HIC-UV/Vis (below 0.1 DAR units). This high variance between injections for the triple quadrupole mass analyzer can in part be attributed to its high sensitivity where any carry-over in the RPLC from previous injection will have a prominent effect on DAR values of subsequent injection, due to a larger variance in the deconvolution profile between runs. This interference was verified when injecting an ADC with a high DAR value after an ADC with a low DAR in which the unconjugated chains peak area gave a noticeably large peak, despite two injections of blank solution was injected in-between (data not shown).

**Fig. 5:**
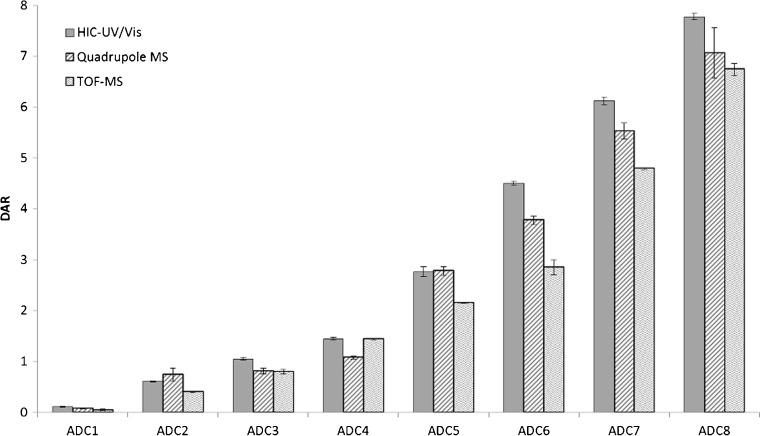
Bar diagram of average DAR value for eight ADC batches with different amounts of conjugated vcMMAE as determined by HIC-UV/Vis, triple quadrupole mass analyzer, or TOF mass analyzer. MS-derived average DAR values are generally slightly lower than HIC-UV/Vis-derived values, especially for higher DAR values. Furthermore, the triple quadrupole mass analyzer in most cases is in higher agreement with HIC-UV/Vis-determined values than the TOF mass analyzer data

As depicted in Fig. 5, the quadrupole-derived average DAR values are in close proximity to HIC-derived values. Surprisingly, for a majority of the batches, DAR values obtained on the triple quadrupole mass analyzer agreed better with HIC-UV/Vis-derived values than was observed on the TOF mass analyzer for the same batch. However, on average, reported DAR value on the HIC-UV/Vis is higher compared with both MS-derived values. Apart from differences derived from the different calculations, as stated above, the following three likely explanations lay in: (i) the payload moiety absorbing light to a certain degree at the monitored wavelength in HIC which may increase the area of the higher conjugation species slightly, relative to the unconjugated trastuzumab; (ii) ionization efficiency which is known to decrease with increasing mass of the analyte leading us to believe there may be a bias in some mass spectra in favor of the lower conjugation species; and (iii) ion suppression of higher conjugation species during co-elution [28]. The extent of these effects on the average DAR value is yet to be determined.

## Conclusions

A triple quadrupole mass analyzer in scan mode can determine Mw of LCs and HCs with different degrees of conjugation originating from heterogeneous batches of ADCs within 2.1 Da of TOF-derived values. Additionally, the obtained spectra made it possible to determine the average DAR value for several batches of cysteine-conjugated ADCs that concurred well with values determined with both TOF mass analyzer and HIC-UV/Vis. Finally, good linear correlation between the TIC response and injected amount for both LCs and HCs was observed over a wide range, indicating that this type of analyzer has the potential to be used in the quantification of intact antibody chains.

## Electronic supplementary material

ESM 1 (PDF 256 kb)
