# Supplementary material for: Application of triple quadrupole mass spectrometry for the characterization of antibody–drug conjugates
Source: Anal Bioanal Chem. 2019 Mar 8;411(12):2569–76. doi: 10.1007/s00216-019-01699-0 (PMC6470114; doi:10.1007/s00216-019-01699-0)
Supplement: Supplementary file 1 — (PDF 256 kb) [file 216_2019_1699_MOESM1_ESM.pdf]

Analytical and Bioanalytical Chemistry

Electronic Supplementary Material

Application of triple quadrupole mass spectrometry for the characterization of antibody–drug conjugates

Malin Källsten, Matthijs Pijnappel, Rafael Hartmann, Fredrik Lehmann, Lucia Kovac, Sara Bergström Lind, Jonas Bergquist

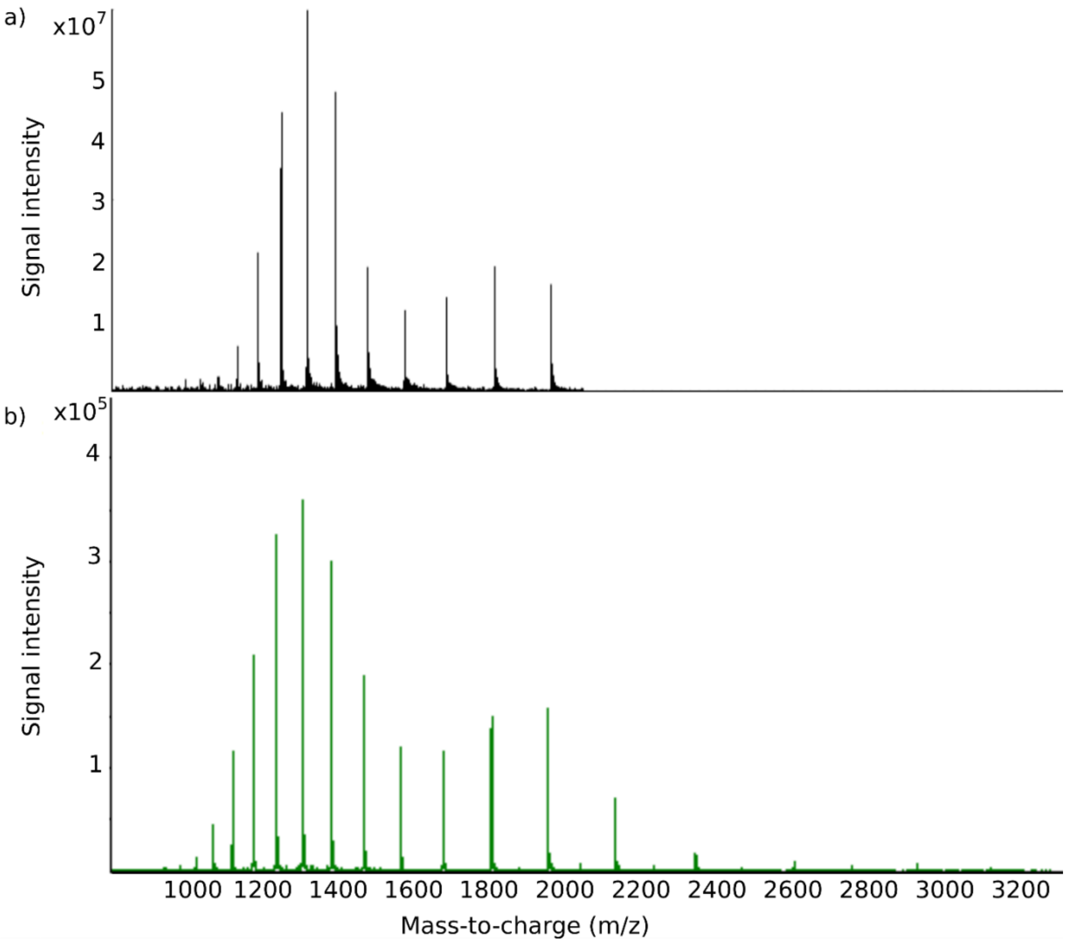

Fig. S1 Mass spectra of unconjugated LC for Trastuzumab as seen on the triple quadrupole mass analyzers (a) and TOF mass analyzer (b). Visualizing that the major part of the charge envelope can be detected on the triple quadrupole mass analyzer, despite the upper mass range being limited to 2040 m/z

Table S1 Evaluation of Mw of LC and HC species for an ADC with an average DAR of 0.1

ADC Chain	Quadrupole mass analyzer Mw (Da)	(RSD [b])	TOF mass analyzer Mw (Da)	(RSD [b])	Difference between techniques (Da)
**LC**	23438.3	(10 ppm)	23440.0	(1 ppm)	1.7
**HC**	49148.6	(12 ppm)	49150.5	(0.4 ppm)	1.9
**HC+1[a]**	50466.7	(12 ppm)	50467.2	(2 ppm)	0.5

[a] The number annotates the number of conjugated one payload-linker moiety to each chain
[b] RSD of triplicate injections

Table S2 Evaluation of Mw of LC and HC species for an ADC with an average DAR of 0.6

ADC Chain	Quadrupole mass analyzer Mw (Da)	(RSD [b])	TOF mass analyzer Mw (Da)	(RSD [b])	Difference between techniques (Da)
**LC**	23438.0	(5 ppm)	23440.0	(1 ppm)	2.0
**LC+1 [a]**	24754.5	(13 ppm)	24756.1	(2 ppm)	1.6
**HC**	49148.4	(6 ppm)	49150.5	(-)	2.1
**HC+1[a]**	50465.4	(5 ppm)	50468.0	(1 ppm)	2.6
**HC+2[a]**	51781.6	(8 ppm)	51784.1	(5 ppm)	2.5

[a] The number annotates the number of conjugated one payload-linker moiety to each chain
[b] RSD of triplicate injections

Table S3 Evaluation of Mw of LC and HC species for an ADC with an average DAR of 1.1

ADC Chain	Quadrupole mass analyzer Mw (Da)	(RSD [b])	TOF mass analyzer Mw (Da)	(RSD [b])	Difference between techniques (Da)
**LC**	23437.8	(3 ppm)	23440.1	(1 ppm)	2.3
**LC+1** [a]	24754.3	(14 ppm)	24756.1	(3 ppm)	1.8
**HC**	49148.2	(2 ppm)	49150.7	(1 ppm)	2.5
**HC+1**[a]	50465.4	(4 ppm)	50468.2	(5 ppm)	2.8
**HC+2**[a]	51780.7	(6 ppm)	51783.8	(6 ppm)	3.2

[a] The number annotates the number of conjugated one payload-linker moiety to each chain
[b] RSD of triplicate injections

Table S4 Evaluation of Mw of LC and HC species for an ADC with an average DAR of 2.8

ADC Chain	Quadrupole mass analyzer Mw (Da)	(RSD [b])	TOF mass analyzer Mw (Da)	(RSD [b])	Difference between techniques (Da)
**LC**	23437.9	(4 ppm)	23439.7	(0.5 ppm)	1.9
**LC+1** [a]	24756.5	(10 ppm)	24756.3	(0.2 ppm)	0.2
**HC**	49147.6	(5 ppm)	49150.3	(1 ppm)	2.8
**HC+1**[a]	50466.2	(3 ppm)	50467.0	(1 ppm)	0.8
**HC+2**[a]	51782.5	(10 ppm)	51783.2	(1 ppm)	0.7
**HC+3**[a]			53100.0	(1 ppm)	-

[a] The number annotates the number of conjugated one payload-linker moiety to each chain
[b] RSD of triplicate injections

Table S5 Evaluation of Mw of LC and HC species for an ADC with an average DAR of 4.5

ADC Chain	Quadrupole mass analyzer Mw (Da)	(RSD [b])	TOF mass analyzer Mw (Da)	(RSD [b])	Difference between techniques (Da)
**LC**	23438.1	(10 ppm)	23439.7	(5 ppm)	1.6
**LC+1** [a]	24756.6	(10 ppm)	24756.4	(5 ppm)	0.2
**HC**	49148.3	(11 ppm)	49149.7	(2 ppm)	1.4
**HC+1**[a]	50466.1	(3 ppm)	50467.4	(4 ppm)	1.3
**HC+2**[a]	51782.3	(11 ppm)	51782.8	(1 ppm)	0.4
**HC+3**[a]			53099.4	(3 ppm)	-

[a] The number annotates the number of conjugated one payload-linker moiety to each chain
[b] RSD of triplicate injections

Table S6 Evaluation of Mw of LC and HC species for an ADC with an average DAR of 6.1

ADC Chain	Quadrupole mass analyzer Mw (Da)	(RSD [b])	TOF mass analyzer Mw (Da)	(RSD [b])	Difference between techniques (Da)
**LC**	23438.3	(2 ppm)	23440.3	(1 ppm)	2.0
**LC+1** [a]	24754.7	(6 ppm)	24756.5	(-)	1.8
**HC**	49148.1	(11 ppm)	49149.4	(18 ppm)	1.3
**HC+1**[a]	50465.0	(6 ppm)	50467.6	(20 ppm)	2.7
**HC+2**[a]	51783.1	(4 ppm)	51785.6	(1 ppm)	2.4
**HC+3**[a]	53101.4	(1 ppm)	53101.7	(0.1 ppm)	0.2

[a] The number annotates the number of conjugated one payload-linker moiety to each chain
[b] RSD of triplicate injections

Table S7 Evaluation of Mw of LC and HC species for an ADC with an average DAR of 7.8

ADC Chain	Quadrupole mass analyzer		TOF mass analyzer		Difference between techniques (Da)
	Mw (Da)	(RSD [b])	Mw (Da)	(RSD [b])	
**LC**	23437.7	(29 ppm)	23440.0	(2 ppm)	2.3
**LC+1** [a]	24754.1	(2 ppm)	24756.5	(1 ppm)	2.4
**HC+2**[a]	51781.5	(10 ppm)	51785.2	(6 ppm)	3.6
**HC+3**[a]	53100.2	(4 ppm)	53101.7	(0.1 ppm)	1.5

[a] The number annotates the number of conjugated one payload-linker moiety to each chain

[b] RSD of triplicate injections
